# Immunohistochemical Evaluation of the Expression of Specific Membrane Antigens in Patients with Pancreatic Ductal Adenocarcinoma

**DOI:** 10.3390/cancers15184586

**Published:** 2023-09-15

**Authors:** Alberto Nicoletti, Federica Vitale, Giuseppe Quero, Mattia Paratore, Claudio Fiorillo, Marcantonio Negri, Angela Carlino, Frediano Inzani, Antonio Gasbarrini, Sergio Alfieri, Lorenzo Zileri Dal Verme

**Affiliations:** 1 Pancreas Unit, CEMAD Centro Malattie dell’Apparato Digerente, Medicina Interna e Gastroenterologia, Dipartimento di Medicina e Chirurgia Traslazionale, Università Cattolica del Sacro Cuore, Fondazione Policlinico Universitario “A. Gemelli” IRCCS, 00168 Rome, Italy; alberto.nicoletti@unicatt.it (A.N.); federica.vitale@guest.policlinicogemelli.it (F.V.); mattia.paratore@guest.policlinicogemelli.it (M.P.); marcantonio.negri@policlinicogemelli.it (M.N.); lorenzo.zileridalverme@policlinicogemelli.it (L.Z.D.V.); 2 Chirurgia Digestiva, Dipartimento di Medicina e Chirurgia Traslazionale, Università Cattolica del Sacro Cuore, Fondazione Policlinico Universitario “A. Gemelli” IRCCS, 00168 Rome, Italy; giuseppe.quero@policlinicogemelli.it (G.Q.); claudio.fiorillo@policlinicogemelli.it (C.F.); sergio.alfieri@unicatt.it (S.A.); 3 Anatomia Patologica, Fondazione Policlinico Universitario “A. Gemelli” IRCCS, 00168 Rome, Italy; angela.carlino@policlinicogemelli.it (A.C.); frediano.inzani@policlinicogemelli.it (F.I.)

**Keywords:** pancreatic cancer, immunohistochemistry, membrane biomarkers, CA 19-9, MUC, Glypican-1, mesothelin, Annexin A10

## Abstract

**Simple Summary:**

Pancreatic ductal adenocarcinoma (PDAC) is an increasing cause of cancer-related death, due to its biologic aggressiveness and the lack of effective treatments. The cell membrane plays a significant role in carcinogenesis, expressing components that mediate the interaction with the peritumoral environment. The aims of our study were to evaluate the expression of six membrane components (CA 19-9, mucin 1 and 4 (MUC1, MUC4), mesothelin (MSLN), Glypican-1 (GPC-1), and Annexin A10 (ANXA10)) on 50 surgical samples of patients with PDAC and correlate it with the oncologic outcomes. The expression was assessed using the histo-score (H-score), a quantitative method based on immunostaining, on tumoral and peritumoral tissues. CA 19-9 and MUC1 showed an intense expression on tumor cells and a lower expression on pancreatic acini and ducts. Moreover, a high intensity of CA 19-9 correlated with a worse prognosis. MUC4, MSLN, GPC-1, and ANXA10 were selectively expressed by PDAC cells and may be potential biomarkers of the disease.

**Abstract:**

(1) Background: Pancreatic ductal adenocarcinoma (PDAC) is one of the most lethal malignancies. The lack of validated disease biomarkers makes timely diagnosis challenging in most cases. Cell membrane and surface proteins play a crucial role in several routes of oncogenesis. The aim of this study was to evaluate the expression of six membrane antigens on PDAC (CA 19-9, mucin 1 and 4 (MUC1, MUC4), mesothelin (MSLN), Annexin A10 (ANXA10), Glypican-1 (GPC-1)) and their correlation with oncologic outcomes. (2) Methods: Immunohistochemical staining for CA 19.9, MUC1, MUC4, MSLN, ANXA10, and GPC-1 of surgical samples of 50 consecutive patients with PDAC was performed. Antigen expression for tumor, ductal, and acinar tissues was classified according to the histo-score (H-score) by two pathologists. (3) Results: Recurrence rate was 47% and 18 patients (36%) deceased (median follow-up 21.5 months). Immunostaining for CA 19-9 and MUC1 showed a significantly higher expression in the neoplastic tissue compared to non-tumor ductal and acinar tissues (*p* < 0.001). MUC4, MSLN, ANXA10, and GPC-1 were selectively expressed in the neoplastic tissue (*p* < 0.001). A CA 19-9 H-score value >270 was independently associated with a worse overall survival (*p* = 0.05) and disease-free survival (*p* = 0.05). (4) Conclusions: CA 19-9 and MUC1 are highly expressed in PDAC cells. The histological expression of CA 19-9 may predict prognosis. MUC4, MSLN, ANXA10, and GPC-1 are selectively expressed by neoplastic tissue and may represent a potential histological biomarker of disease.

## 1. Introduction

Pancreatic ductal adenocarcinoma (PDAC) is one of the major public health concerns, due to its increasing incidence, the absence of effective therapies, and the lack of reliable early diagnostic markers [1].

Indeed, the global incidence of PDAC currently ranges from 1 to 10 cases per 100,000, representing the seventh-most lethal disease and the twelfth most prevalent cancer worldwide [2]. Furthermore, according to current epidemiological evidence, PDAC incidence is expected to more than double in the near future [3], becoming the second leading cause of cancer-related death in the next ten years [4]. In terms of long-term outcomes, PDAC is notably characterized by a dismal prognosis with a reported 5-year survival of only 8–9% [5]. This is mainly due to a late diagnosis (generally at a locally advanced/metastatic stage) as compared to other gastrointestinal tumors, leading to a low percentage of patients that can benefit from a curative surgical treatment.

All these characteristics inevitably make PDAC one of the major challenging oncological diseases, both for physicians and researchers; thus, it is crucial to identify sensitive and specific biomarkers for the early detection, targeted treatment, and prognostic assessment of PDAC.

Currently, serum carbohydrate antigen 19-9 (CA 19-9) is the most studied membrane antigen and is the only biomarker approved by the Food and Drug Administration (FDA) in the management of PDAC [6,7,8]. However, the low sensitivity, specificity, and positive predictive value of serum CA 19-9 do not allow for its use as a screening biomarker [9], while it is efficiently used as a prognostic tool for the evaluation of tumor response in case of neoadjuvant chemotherapy [10].

In this challenging process, immunohistochemistry (IHC) is a broadly available technique for tissue diagnostics, particularly in the field of oncology, and a platform for biomarker detection. Indeed, it is a sensible, reliable, and cost-effective tool to evaluate the expression of pancreatic cancer antigens. It is based on antibody affinity to tissue antigens, commonly within formalin-fixed paraffin-embedded tissue samples. One of the major advantages of IHC is its availability worldwide, which allows for a wide application of its use [11,12].

The cell membrane hosts several surface receptors, transport proteins, enzymes, and adhesion molecules, whose expression and activity are altered in cancer cells [13].

Moreover, these proteins play a remarkable role in the interaction with the complex tumor microenvironment which influences the cell proliferation, invasion, metastasis, and abnormal immunology associated with tumors [14,15].

Hence, it is implicit that the early detection of these membrane components via the means of immunohistochemical studies could significantly support the physician in a prompt diagnosis of PDAC. For this purpose, several membrane antigens have already been investigated and proposed as potential biomarkers.

Some evidence has supported the potential use of Glypican-1 (GPC1) [16] thanks to its overexpression in PDAC [17,18]. Similarly, both mucins (MUC) 1 and 4 have been considered potentially valuable biomarkers, whose overexpression correlates to a poorer prognosis of PDAC. A reliable specificity and sensitivity have also been reported for mesothelin (MSLN) [19] and Annexin A10 (ANXA10) [20,21].

Despite all this evidence, the limited number of patients enrolled in these studies significantly limit the generalization of the results. Moreover, only a few authors have compared the intensity of the expression of specific membrane components between neoplastic and normal pancreatic tissue, in order to define those biomarkers selectively expressed by PDAC.

Therefore, the aim of the present study is to investigate the expression of six membrane antigens, namely CA 19-9, GPC1, MUC1, MUC4, MSLN, and ANXA10, in tumoral and peritumoral pancreatic tissues in order to define their potential role as specific PDAC biomarkers and to investigate the potential relationship between their grade of expression and the oncologic outcomes.

## 2. Material and Methods

### 2.1. Study Population

After Institutional Review Board (IRB) approval (study protocol ID 3666, protocol 0049970/20 date of approval 10 December 2020), 50 consecutive patients who underwent pancreatic resection for a proven diagnosis of PDAC at the Fondazione Policlinico Universitario “Agostino Gemelli” IRCCS of Rome, between January 2018 and May 2020, were prospectively enrolled in the study. Neoadjuvant therapy or previous history of other malignancies were defined as exclusion criteria.

Demographic, clinical, laboratory, operative, and radiological data were collected from hospital records to develop prospectively maintained databases. Specifically, age, sex, medical history of diabetes, smoking, familiarity, associated history of chronic pancreatitis, tumor location (head, body, or tail of the pancreas), tumor staging (pTNM) were also recorded and assessed according to the 8th edition of the AJCC guidelines [22]. Follow-up data were collected from oncological records and included recurrence rate, tumor-related mortality at the last follow-up, disease free-survival (DFS), and overall survival (OS).

The study protocol conforms to the guidelines of the 1975 Declaration of Helsinki.

### 2.2. Histopathology

All histopathological samples of the enrolled patients in the form of formalin-fixed paraffin-embedded tissue blocks were obtained from the archives of the Department of Pathology. Deparaffinized sections (5 µm) were rehydrated via passing them through decreasingly concentrated ethyl alcohol solutions and then washed with xylene. Then, the slices were stained with hematoxylin and eosin.

### 2.3. Immunohistochemistry

The immunohistochemical staining to study the membrane antigens was performed using monoclonal antibody directed against each specific antigen: CA 19-9 (clone C241:5:1:4, Leica Biosystems, Newcastle, UK), MUC1 (clone MA695, Leica Biosystems, Newcastle, UK), MUC4 (clone EPR9308, Leica Biosystems, Newcastle, UK), MSLN (clone 5B2, Leica Biosystems, Newcastle, UK), ANXA10 (Clone EPR 19507, Abcam Inc., Toronto, ON, Canada), and GPC1 (Clone EPR 22580-72, Abcam Inc., Toronto, ON, Canada). A BOND Polymer Refine Detection System and a 20 min high pH (=9)-revealing procedure were performed for GPC1, MUC1, MUC4, MSLN, and ANXA10. As for CA 19-9, the same procedure was performed at low pH (=6) for 10 min.

### 2.4. Histopathology Analysis and Histo-Score (H-Score)

Two blinded pathologists, who were experts in pancreatic cancer pathology, evaluated the expression of the membrane antigens of anonymized preparations using an optical microscope in the absence of further clinical and pathology information, such as grading, staging, biochemical tests.

The scoring of each antigen was determined by calculating the histo-score (H-score) using a semi-quantitative method combining immunostaining intensities and percentages of positive cancer cells.

Immunostaining intensity was defined as: 3+ for an intense (strong) reaction that was easily visible even at low magnification (4×), 2+ for a moderate reaction that was visible at low magnifications (4×), 1+ for a weak reaction that was mainly visible at high magnifications (from 20× to 40×), 0 in case of absence of visible reaction at high magnifications (40×). The percentage of positive tumor cells (estimated to the closest 10) displaying the specified staining intensities (3+, 2+, 1+, 0) was measured in relation to all visible tumor cells on each tissue sample, and always summed up to 100% malignant cells. The H-score was calculated by summing the multiplication product of the staining intensity and the percentage of cells showing the same intensity, according to the following formula [23,24]:

H-score = 0 (%cells 0) + 1 (%cells 1+) + 2 (%cells 2+) + 3 (%cells 3+)(1)

H-score ranges from 0 to 300. Numerical H-score was also classified into: 0 category (absent) when the result of the formula is 0, 1+ (weak and focal) when ranging from 1 to 50, 2+ (moderate) when ranging from 51 to 150, 3+ (intense and widespread) when ranging from 151 to 300 [25].

### 2.5. Statistics Analysis

SPSS version 27.0 (IBM Corp., Armonk, NY, USA) was used for data analysis. All continuous data were reported as median and interquartile range (IQR) while numbers and percentages were used for all categorical data. The correlation between categorical variables was assessed with Chi-squared tests. The Kruskal–Wallis test was used to compare the antigen expression between neoplastic and normal pancreatic tissue. Correlation analyses were performed via linear regression and binary logistic regression. Kaplan–Meier curves were constructed by using the median H-score value of each antigen as the cut-off. For all tests, a *p*-value ≤ 0.05 was considered statistically significant.

## 3. Results

### 3.1. Clinical and Demographic Characteristics of the Study Population

Fifty patients with a histopathologic-proven diagnosis of PDAC who underwent surgery with a curative intent between January 2018 and May 2020 were prospectively enrolled. The clinical and demographic characteristics of the population and the prevalence of risk factors for PDAC are shown in Table 1.

As reported in Table 2, the head of the pancreas was the most frequent location of PDAC (41 patients, 82%), followed by the body (6 patients, 12%) and tail (3 patients, 6%) of the pancreas. In terms of pathological staging, the majority of patients presented a T2 lesion (42 patients, 84%) and lymph node metastases (N1: 23 patients, 46%; N2: 14 patients, 28%), thus identifying stages IIB and III as the most frequent in the study cohort (46% and 28%, respectively). In terms of tumor grading, an intermediate differentiation was documented in 88% of the study population (44 patients). After surgery, all patients underwent adjuvant chemotherapy with nab-paclitaxel and gemcitabine.

### 3.2. Antigen Expression in the Neoplastic Tissue

**Figure 1:**
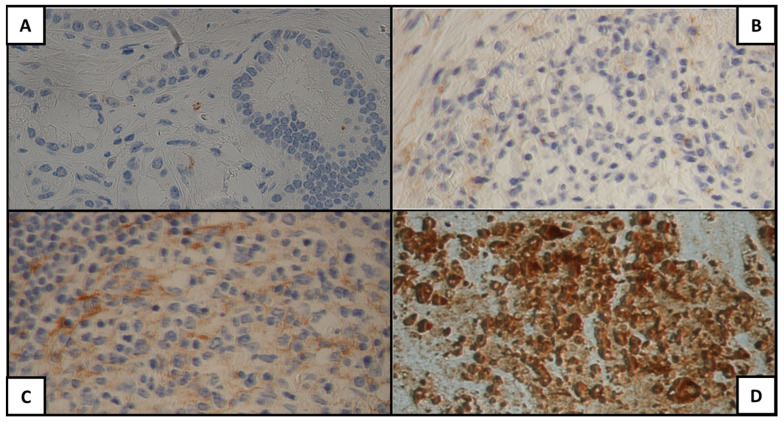
MUC1 expression for each H-score category: (**A**) 0, (**B**) 1+, (**C**) 2+, (**D**) 3+.

**Figure 2:**
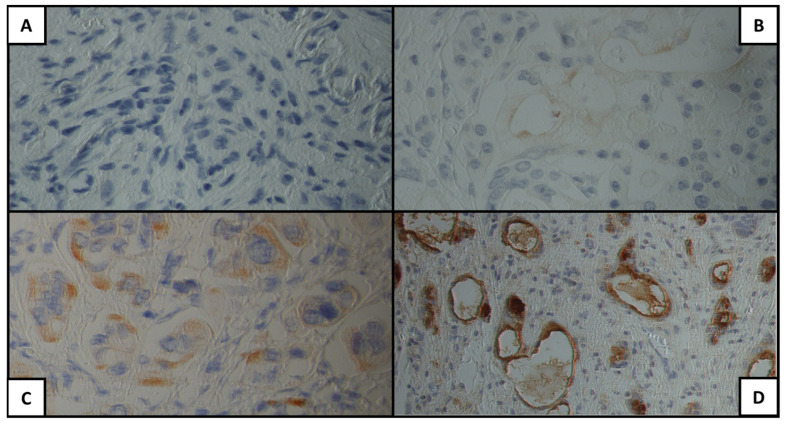
Mesothelin expression for each H-score category: (**A**) 0, (**B**) 1+, (**C**) 2+, (**D**) 3+.

**Figure 3:**
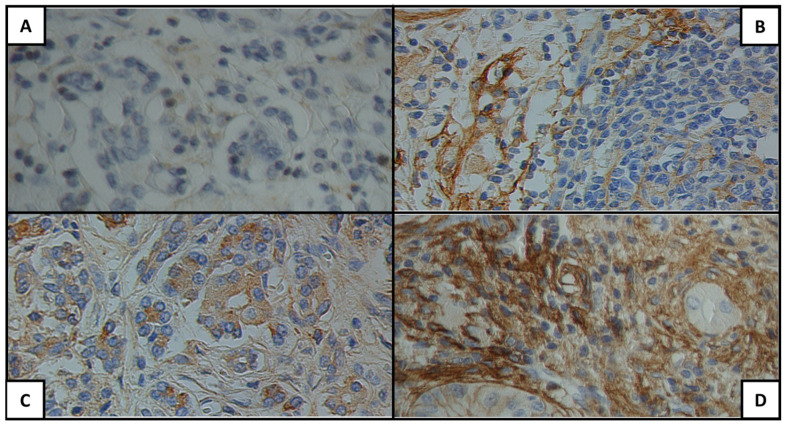
Glypican-1 expression for each H-score category: (**A**) 0, (**B**) 1+, (**C**) 2+, (**D**) 3+.

Figure 1, Figure 2 and Figure 3 show examples of the antigen expression for each H-score category. Tumor grading and staging and antigen H-score for each patient are presented in Supplementary Material Table S1.

In the neoplastic tissue, CA 19-9 was expressed at a high intensity (H-score 3+) in 86% of cases (43 patients), while a low (H-score 1+) and moderate (H-score 2+) intensity was observed in the remaining patients (4% and 10% of the study cohort, respectively).

Although lower than CA 19-9, a significant high intensity (H-score 3+) was also evidenced for MUC 1 (38 patients, 76%), while a low (H-score 1+) or intermediate (H-score 2+) intensity was documented in 8% (4 patients) and 14% (7 patients) of cases, respectively. No expression of MUC1 was observed in only 1 patient (2%).

MUC4 staining demonstrated a high intensity (H-score 3+) in 40% of cases (20 patients), a moderate intensity (H-score 2+) in 36% of the study population (18 patients), and a low intensity (H-score 1+) in 24% of cases (12 patients).

Similarly, MSLN was expressed at a high intensity (H-score 3+) in 44% of cases (22 patients), at an intermediate intensity (H-score 2+) in 40% (20 patients), and at a low intensity (H-score 1+) in 14% of cases (7 patients).

ANXA10 staining showed a high intensity (H-score 3+) in 44% of cases (22 patients) and a low (H-score 1+) or intermediate (H-score 2+) intensity in 40% and 14%, respectively.

Both MSLN and ANXA10 were not detectable (H-score 0+) in only 2% of specimens (1 patient each).

Finally, GPC1 was intensively expressed (H-score 3+) in 8% of the analyzed samples (4 patients), moderately expressed (H-score 2+) in 40% (20 cases), and lowly expressed (H-score 1+) in 48% (24 cases). It was not evidenced (H-score 0+) in 4% of the population (2 patients).

### 3.3. Comparison of the Antigen Expression between Neoplastic and Non-Neoplastic Tissues

To compare the antigen expression between neoplastic and non-neoplastic pancreatic tissues, the H-score for the antigen expression was also assessed on non-neoplastic pancreatic ducts and acini of the same tissue samples, as shown in Table 3.

**Figure 4:**
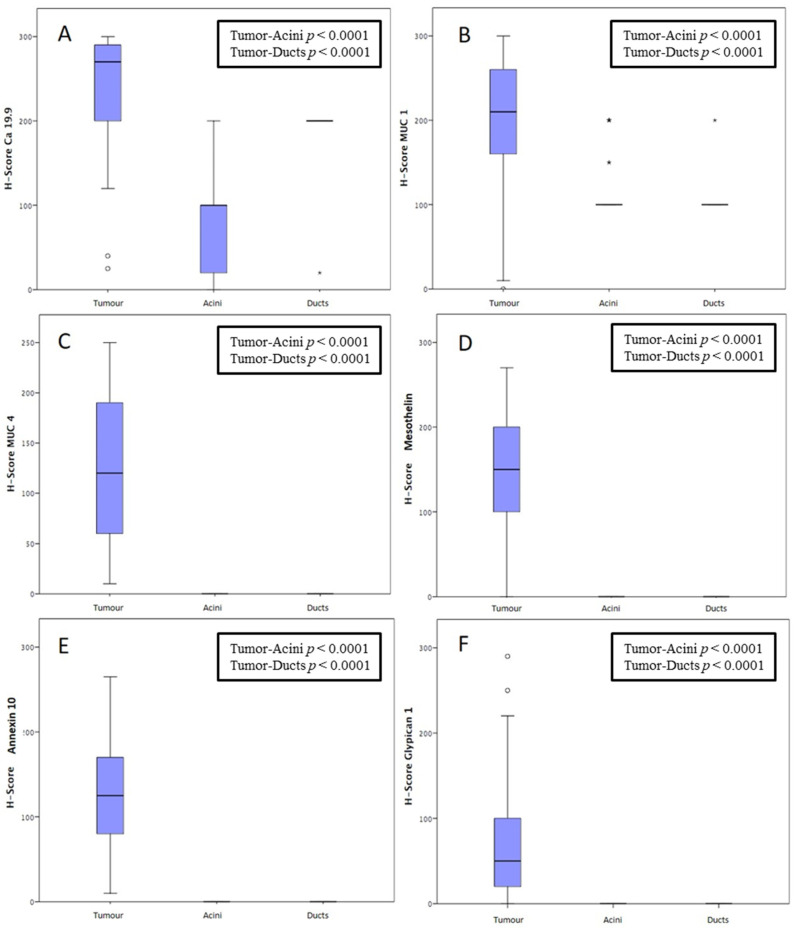
Comparison of antigen expression between tumor, acini, and ducts: (**A**) CA 19-9, (**B**) MUC1, (**C**) MUC4, (**D**) mesothelin, (**E**) Annexin A10, (**F**) Glypican-1.

Figure 4 presents the statistical comparison of each antigen expression in the neoplastic tissue, pancreatic ducts, and acini according to the H-score value. The median CA 19-9 H-score value of the neoplastic tissues was 270 (IQR: 200–292.5). Similarly, the ducts showed a high expression of this antigen, with a median H-score of 200 and a homogeneous distribution among the different samples (IQR: 200–200). Conversely, CA 19-9 acinar expression was very heterogeneous among different samples, although significantly less intense as compared to the tumor and ductal expression. Indeed, for pancreatic acini, the median CA 19-9 H-score value was 100, with an IQR comprised between 20 and 100. The difference in the CA 19-9 expression among the neoplastic tissue, pancreatic ducts, and acini resulted in being statistically significant (*p* < 0.0001).

As for MUC1, the median tumor H-score value was 210 (IQR: 157.5–260). Both the pancreatic ducts and acini showed a homogeneous intermediate H-score (100, IQR 100–100), with a statistically significant difference compared with the neoplastic tissue (*p* < 0.0001).

Although staining for MUC4 showed a lower absolute H-score value than MUC 1 (120, IQR: 58.75–190), no expression (H-score 0) was found in the pancreatic ducts and acini (*p* < 0.0001).

Similarly, no MSLN, ANXA10, and GPC1 expression was observed in the non-neoplastic tissues. The median MSLN, ANXA10, and CPC1 H-score values were 150 (IQR: 100–205), 125 (IQR: 77.5–172.5), and 50 (IQR: 20–100), respectively.

### 3.4. Clinical Outcomes and Correlation with Antigen Expression

The median follow-up time of the study population was 21.5 months (mean 23.1 months). During the follow-up period, PDAC recurred in 23 patients (46%), with a median DFS of 15 months. In total, 18 patients (36%) deceased with a median OS of 21.5 months.

Antigen expression in the neoplastic tissue, in terms of H-score, was correlated with oncologic outcomes, such as DFS and OS. In the univariate analysis, both a CA 19-9 H-score >270 and >150 were inversely associated with OS (*p* = 0.05 and *p* = 0.02, respectively). In the multivariate analysis, both these variables were also independently associated with OS (OR: 2.7, CI 95%: 1.7–9.6, *p* = 0.02; OR: 3.1, CI 95%: 1.4–7.6, *p* = 0.01). Conversely, no correlation was found between the expression of MUC1, MUC4, MSLN, ANXA10, and GPC1 and OS. Instead, the expression of MUC1, MUC4, MSLN, ANXA10, and GPC1 was not associated with OS (Supplementary Material Table S2, Supplementary Material Figure S1).

Interestingly, in the univariate analysis, a CA 19-9 H-score > 270 was associated with a worse DFS (*p* = 0.05) (Supplementary Material Table S2 and Supplementary Material Figure S2). However, this parameter was not confirmed as an independent predictor of DFS in the multivariate analysis (Supplementary Material Table S2).

## 4. Discussion

PDAC is widely recognized as one of the most lethal oncological diseases, with the number of cases and deaths both estimated to increase by 40% by 2035 [4]. Although multimodal treatment strategies seem to have positively influenced the PDAC clinical course, a significant portion of patients is still diagnosed at an advanced and untreatable stage. This inevitably emphasizes the requirement for novel diagnostic strategies to support earlier diagnosis and consequent treatment.

In this field, the examination of membrane antigens in PDAC has progressively gained interest. The potential ability to recognize specific disease biomarkers would bring several advantages. Firstly, it would allow for a precise histopathologic diagnosis. Secondly, it may be the basis for the further development of non-invasive diagnostic tests. Indeed, the cell membrane is involved in several processes of neoplastic cells, such as the communication between them and the tumor microenvironment, inhibition of immune cells, and formation of the metastatic niche [26]. The intense trafficking of tumor cell membrane often determines an increased formation of extracellular vesicles, which host antigens of the producing cell and have recently been considered as promising biomarkers for an easier and earlier diagnosis of several malignancies [27,28].

The advent of advanced and molecular techniques has certainly upgraded the ability to identify tissue biomarkers. However, these techniques are affected by high costs and the need for significant expertise and fresh samples. These considerations limit the application of the results to clinical practice. On the other hand, IHC does not allow for a quantitative estimation of antigens, but it is still considered as a reliable technique to evaluate potential biomarkers in clinical patient cohorts [11,12,29]. Hence, in order to favor the clinical application of the results, we designed a study based on IHC, which is broadly available in most pathology laboratories worldwide.

Therefore, the aim of our study was to examine the expression of six membrane antigens in PDAC and normal pancreatic tissue in order to define the more precise and selective membrane antigen expressed by tumor cells, and to evaluate the correlation between them and the oncologic outcomes. To our knowledge, this is the first study to contemporarily analyze the expression of six membrane antigens for the diagnosis of PDAC on one of the largest cohorts of patients in the literature.

The study population had similar demographic and clinical characteristics, as compared with other studies. However, despite a high prevalence of patients with moderately advanced pathologic staging (IIB stage: 46%; III stage: 28%) at the time of surgery, patients presented better prognostic outcomes [30,31]. No patient died in the early post-operative period. These results may be due to the fact that the study was conducted in a tertiary care university hospital with a high-volume digestive surgery for pancreatic malignancies, which is strictly integrated with the medical oncology unit [32,33].

According to our results, MUC4, MSLN, ANXA10, and GPC1 showed an evident expression in the vast majority of the neoplastic samples (range 96–100%), while no expression was noted in all the normal pancreatic tissues analyzed. Therefore, they may represent promising biomarkers for the immunohistochemical diagnosis of PDAC, as no validated antigen target is currently available for this purpose.

The potential diagnostic role of MSLN was already reported by other authors, who demonstrated a selective expression with high prevalence in PDAC cells [34]. This demonstrates not only the diagnostic potential of this antigen, but also its therapeutic potential. Indeed, MSLN-based immunotherapies have been proposed for different MSLN-expressing malignancies, such as PDAC itself, ovarian cancer, malignant mesothelioma, and lung adenocarcinoma [35,36]. However, in our population, we did not evidence any correlation between MSLN expression and clinical and pathologic characteristics and oncologic outcomes, as previously demonstrated by other authors [34].

Similarly, MUC4 was also selectively expressed in neoplastic tissues with an H-score ≥ 1+ in 100% of cases and an H-score ≥ 2+ in 76%, while no evidence was encountered in pancreatic ducts and acini. These rates of expression are in line with previous reports (even though in smaller cohorts of patients), according to which, MUC4 expression ranged between 78% and 89% of PDAC tissues [37,38,39]. In other experiences, the expression of MUC4 was also associated with chemoresistance and correlated negatively with prognosis [40,41]. Of note, similarly to MSLN, MUC4 was recently considered as a valuable tumor-related target for immunotherapy and a potential candidate vaccine for PDAC, thanks to its selective expression on PDAC cells [18].

Compared to other studies, the prevalence of ANXA10 expression was significantly higher in our population, reaching a selective expression in PDAC cells in up to 98% of samples, while it was absent in both acini and ducts cells. Although even for ANXA10, no association was found between its expression and long-term outcomes, probably due to our short follow-up [23,42]. ANXA10 has been recognized as a valuable diagnostic tool in differentiating liver PDAC metastases from cholangiocarcinoma [21] or other malignancies, with a sensitivity and specificity of 83% and 95%, respectively [22].

As for GPC1, multiple authors have already documented its high sensitivity and specificity in identifying PDAC [17], making it a further potential specific serum antigen for diagnosis. Indeed, cell expression was confirmed in 96% of the PDAC samples in our cohort, as compared to a complete absence in normal pancreatic tissues.

A particular mention is needed for CA 19-9. Despite the short follow-up of our study cohort, in our population, the neoplastic tissue CA 19-9 H-score correlated with oncologic outcomes. In particular, an H-score >150 was associated with a 3.1 higher risk of worse OS compared with patients with lower H-score values (CI 95% 1.4–7.6, *p* = 0.01). Similar to other studies [43,44], high CA 19-9 levels (cut-off 157 UI/mL) were associated with a worse prognosis in terms of OS (OR 1.9, CI 95% 1–5.9, *p* = 0.02).

Despite a statistically significant difference between neoplastic and paracancerous tissues, MUC1 was not selectively expressed by tumor cells. Hence, it is not adequate for the immunohistochemical diagnosis of PDAC.

The present study has some limitations. Firstly, there was no control population with benign pancreatic disease. However, patients with benign non-precancerous pancreatic disease rarely undergo surgery. Moreover, the disproportion of the sample amounts would significantly limit a comparison with fine-needle aspiration biopsy samples. Secondly, we did not analyze antigen expression in other gastrointestinal malignancies and tissue samples from other organs. However, PDAC and cholangiocarcinoma demonstrated comparable features in terms of tumor biology and prognosis [45]. At present, surgical and oncological management are similar for PDAC and cholangiocarcinoma. As for other gastrointestinal malignancies, the anatomical site of the tumor origin and standard pathology are sufficient to distinguish different entities [46]. In addition, we did not compare the IHC results with other advanced and molecular techniques, such as Western blotting, RNA sequencing, and mass spectrometry data. Finally, the small population size is another limit of the study; nevertheless, it is in line with the majority of immunohistochemistry studies.

## 5. Conclusions

In conclusion, among the antigens investigated in this study, MUC4, MSLN, ANXA10, and GPC1 emerge as promising selective disease biomarkers. On the other hand, CA 19-9 may be more useful as a prognostic biomarker of the disease.

To confirm these findings, further studies including a larger sample size and control groups affected by benign pancreatic diseases, other malignancies, and tissue samples from other organs would be required. Indeed, it is crucial to meet the absolute need to validate specific antigens for the differential diagnosis between PDAC, other neoplasms, and benign pancreatic conditions. Furthermore, the identification of such biomarkers may guide the evaluation of a non-invasive diagnostic method for the detection of PDAC at an early stage.

## Figures and Tables

**Table 1:** Clinical and demographic characteristics of the study population.

Characteristic	Median (IQR)/Patients n (%)
Age (years)	67.5 (57.7–72.2)
Sex	M: 31 (62); F: 19 (38)
Diabetes mellitus	14 (28)
Smokers	32 (64)
BMI > 25 kg/m^2^	9 (18)
Familiar history	17 (34)
Chronic pancreatitis	1 (2)

Abbreviations: BMI, body mass index; F, females; IQR, interquartile range; M, males.

**Table 2:** Histopathology data of the study population.

Tumor Site	Patients n (%)
Head Body Tail	41 (82) 6 (12) 3 (6)
**T**	**Patients n (%)**
1 2 3 4	3 (6) 42 (84) 5 (8) 0 (0)
**N**	**Patients n (%)**
0 1 2	13 (26) 23 (46) 14 (28)
**Tumor Stage**	**Patients n (%)**
IA IB IIA IIB III IV	3 (6) 10 (20) 0 (0) 23 (46) 14 (28) 0 (0)
**Grading**	**Patients n (%)**
G1 G2 G3	1 (2) 44 (88) 5 (10)

**Table 3:** H-score categories according to the expression of CA 19-9, MUC1, MUC4, MSLN, ANXA10, and GPC-1 on neoplastic tissue, acini, and pancreatic ducts.

CA 19-9 Tumor	Patients n (%)	CA 19-9 Acini	Patients n (%)	CA 19-9 Ducts	Patients n (%)
H-score 0 H-score 1+ H-score 2+ H-score 3+	0 (0) 2 (4) 5 (10) 43 (86)	H-score 0 H-score 1+ H-score 2+ H-score 3+	10 (20) 6 (12) 25 (50) 9 (18)	H-score 0 H-score 1+ H-score 2+ H-score 3+	0 (0) 1 (2) 0 (0) 49 (98)
**MUC1** **Tumor**	**Patients** **n (%)**	**MUC1** **Acini**	**Patients** **n (%)**	**MUC1** **Ducts**	**Patients** **n (%)**
H-score 0 H-score 1+ H-score 2+ H-score 3+	1 (2) 4 (8) 7 (14) 38 (76)	H-score 0 H-score 1+ H-score 2+ H-score 3+	0 (0) 0 (0) 44 (88) 6 (12)	H-score 0 H-score 1+ H-score 2+ H-score 3+	0 (0) 0 (0) 49 (98) 1 (2)
**MUC4** **Tumor**	**Patients** **n (%)**	**MUC4** **Acini**	**Patients** **n (%)**	**MUC4** **Ducts**	**Patients** **n (%)**
H-score 0 H-score 1+ H-score 2+ H-score 3+	0 (0) 12 (24) 18 (36) 20 (40)	H-score 0 H-score 1+ H-score 2+ H-score 3+	50 (100) 0 (0) 0 (0) 0 (0)	H-score 0 H-score 1+ H-score 2+ H-score 3+	50 (100) 0 (0) 0 (0) 0 (0)
**MSLN** **Tumor**	**Patients** **n (%)**	**MSLN** **Acini**	**Patients** **n (%)**	**MSLN** **Ducts**	**Patients** **n (%)**
H-score 0 H-score 1+ H-score 2+ H-score 3+	1 (2) 7 (14) 20 (40) 22(44)	H-score 0 H-score 1+ H-score 2+ H-score 3+	50 (100) 0 (0) 0 (0) 0 (0)	H-score 0 H-score 1+ H-score 2+ H-score 3+	50 (100) 0 (0) 0 (0) 0 (0)
**ANXA10** **Tumor**	**Patients** **n (%)**	**ANXA10** **Acini**	**Patients** **n (%)**	**ANXA10** **Ducts**	**Patients** **n (%)**
H-score 0 H-score 1+ H-score 2+ H-score 3+	1 (2) 7 (14) 20 (40) 22(44)	H-score 0 H-score 1+ H-score 2+ H-score 3+	50 (100) 0 (0) 0 (0) 0 (0)	H-score 0 H-score 1+ H-score 2+ H-score 3+	50 (100) 0 (0) 0 (0) 0 (0)
**GPC1** **Tumor**	**Patients** **n (%)**	**GPC1** **Acini**	**Patients** **n (%)**	**GPC1** **Ducts**	**Patients** **n (%)**
H-score 0 H-score 1+ H-score 2+ H-score 3+	2 (4) 24 (48) 20 (40) 4 (8)	H-score 0 H-score 1+ H-score 2+ H-score 3+	50 (100) 0 (0) 0 (0) 0 (0)	H-score 0 H-score 1+ H-score 2+ H-score 3+	50 (100) 0 (0) 0 (0) 0 (0)

## Data Availability

The data presented in this study are available on reasonable request from the corresponding author.

## Supplementary Materials

The following supporting information can be downloaded at: https://www.mdpi.com/article/10.3390/cancers15184586/s1, Figure S1: Kaplan-Meier curve of overall survival (OS) according to CA 19-9 H-score (cut-off 270); Figure S2: Kaplan-Meier curve of disease-free survival (DFS) according to CA 19-9 H-score (cut-off 270); Table S1: Tumor grading and staging and antigen H-score for each patient; Table S2: Univariate and multivariate analysis to evaluate the correlation between OS and DFS and clinical-demographic and immunohistochemical factors.
