# Supplementary material for: Immunohistochemical Evaluation of the Expression of Specific Membrane Antigens in Patients with Pancreatic Ductal Adenocarcinoma
Source: Cancers (Basel). 2023 Sep 15;15(18):4586. doi: 10.3390/cancers15184586 (PMC10526869; doi:10.3390/cancers15184586)
Supplement: Supplementary file 1 [file cancers-15-04586-s001.zip › cancers-2509994-supplementary.pdf]

Supplementary material Table S1. Tumor grading and staging and antigen H-score for each patient.

Patient	Grading	T	N	Staging	CA 19-9 H-score	MUC1 H-score	MUC4 H-score	MSLN H-score	ANXA10 H-score	GPC1 H-score
1	G2	T2	N1	IIB	300	290	60	250	90	220
2	G2	T2	N1	IIB	300	280	190	110	195	20
3	G3	T2	N1	IIB	280	220	100	250	170	50
4	G2	T2	N2	III	300	180	30	270	100	20
5	G2	T2	N2	III	260	190	90	50	10	0
6	G2	T2	N2	III	120	160	250	110	165	120
7	G2	T2	N1	IIB	290	260	80	220	150	10
8	G2	T2	N0	IB	200	210	110	50	65	20
9	G2	T2	N2	III	200	175	70	25	170	10
10	G2	T2	N2	III	140	210	240	240	65	150
11	G2	T3	N1	IIB	280	260	15	150	170	10
12	G2	T2	N0	IB	270	210	50	130	180	10
13	G2	T2	N0	IB	290	290	190	165	240	50
14	G2	T2	N1	IIB	260	130	170	235	160	110
15	G2	T2	N1	IIB	280	150	110	165	135	80
16	G2	T1	N0	IA	300	290	10	150	100	40
17	G2	T2	N1	IIB	250	260	210	200	200	30
18	G3	T2	N1	IIB	260	215	240	220	60	80
19	G3	T3	N2	III	290	190	65	240	110	70
20	G2	T3	N1	IIB	170	200	195	260	85	65
21	G2	T2	N2	III	220	230	140	140	170	5
22	G2	T2	N2	III	300	230	165	170	115	140
23	G2	T2	N2	III	270	260	220	110	200	120
24	G2	T2	N1	IIB	270	220	160	110	265	70
25	G2	T3	N1	IIB	200	280	190	260	70	150
26	G2	T2	N1	IIB	250	240	110	50	265	70
27	G2	T2	N2	III	280	260	190	220	115	20
28	G3	T2	N2	III	25	280	95	85	110	30
29	G3	T2	N1	IIB	280	300	40	0	10	290
30	G2	T2	N0	IB	170	210	70	200	95	50
31	G2	T1	N0	IA	140	215	215	175	70	190

32	G2	T2	N1	IIB	270	50	130	90	110	0
33	G2	T2	N2	III	120	30	40	150	150	50
34	G2	T2	N1	IIB	160	180	140	100	120	80
35	G2	T2	N1	IIB	280	220	110	70	210	20
36	G2	T2	N1	IIB	270	180	55	190	190	100
37	G2	T2	N0	IB	40	40	70	230	110	40
38	G2	T2	N2	III	280	110	160	160	200	50
39	G2	T2	N0	IB	220	200	20	30	55	250
40	G2	T2	N1	IIB	280	10	20	70	140	60
41	G2	T2	N1	IIB	150	0	10	100	10	40
42	G2	T2	N0	IB	300	120	170	110	190	20
43	G2	T2	N0	IB	300	150	40	160	45	60
44	G2	T2	N1	IIB	300	110	200	135	130	80
45	G2	T3	N2	III	300	130	50	150	15	60
46	G2	T2	N0	IB	300	180	180	110	140	20
47	G2	T2	N1	IIB	300	230	140	110	230	100
48	G2	T2	N0	IB	210	280	190	190	55	130
49	G2	T2	N1	IIB	300	240	180	40	150	40
50	G1	T1	N0	IA	210	220	140	130	80	20

Abbreviations: ANXA10, Annexin A10; CA 19-9, carbohydrate antigen 19-9; GPC-1, Glypican1; MSLN, mesothelin; MUC1, mucin 1; MUC4, mucin 4.

Supplementary material Table S2. Univariate and multivariate analysis to evaluate the correlation between OS and DFS and clinical-demographic and immunohistochemical factors.

**Variable**	**Univariate Analysis**				**Multivariate Analysis**					
	**5-year OS (%)**	**p**	**5-year DFS (%)**	**p**	**5-year OS**			**5-year DFS**		
					**OR**	**95% CI**	**p**	**OR**	**95% CI**	**p**
Age, ≤65/>65	59.8/55.3	0.81	56.4/46.6	0.72	**-**	**-**	**-**	**-**	**-**	**-**
T stage, I-II/III-IV	66.7/53.5	0.84	53.3/33.3	0.35	-	-	-	-	-	-
N, 0-1/2-3	68.3/31.3	**0.02**	62.8/26.8	**0.01**	2.1	1.5-3.7	**0.02**	1.9	1.19-4.9	**0.03**
Stage, I-II/III-IV	63/42.2	0.34	60.6/28.6	**0.04**	-	-	-	2.12	1.45-9.9	**0.02**
Neutrophils, ≤4.8/>4.8x10^9/l	47.8/39.5	**0.02**	64.3/25.9	**0.04**	2.9	1-7.9	**0.04**	2.2	0.98-5	**0.05**
Lymphocites, ≤1.7/>1.7x10^9/l	66.6/48.3	0.35	50/51.2	0.92	-	-	**-**	-	-	-
Neutrophils/Lymphocites, ≤3.7/>3.7	58.6/66.7	0.88	41.7/66.7	0.46	-	-	-	-	-	-
Amylase, ≤72/>72	75.1/33.9	**0.05**	55.3/39.6	0.38	1.7	1-7.5	**0.05**	-	-	-
Lipase, ≤106/>106	56.9/61.4	0.84	47.2/43.6	0.72	-	-	-	-	-	-
AST, ≤175/>175	53.1/61.6	0.7	58.2/38.1	0.22	-	-	-	-	-	-
ALT, ≤107/>107	51.4/55.6	0.77	48.8/49	0.54	-	-	-	-	-	-
Phosphatase, ≤115/>115	54.5/53.1	0.72	68.2/38.3	0.11	-	-	-	-	-	-
Gamma GT, ≤105/>105	69.7/45.3	0.25	62.3/37.5	0.14	-	-	-	-	-	-
Bilirubin tot, ≤2.8/>2.8	65.1/44.9	0.43	58.4/39.6	0.34	-	-	-	-	-	-
CEA, medium ≤2.8/>2.8	59.8/50	0.17	50.5/53.6	0.78	-	-	-	-	-	-
CA 19-9 serum, median ≤157/>157	67.1/32.1	**0.03**	62.2/46.4	0.14	1.9	1-5.9	**0.02**	-	-	-
CA 19-9, HS median, ≤270/>270	60.6/47.2	**0.05**	58.7/42.5	**0.05**	2.7	1.7-9.6	**0.02**	1.9	0.6-5.7	0.23
CA 19-9, HS≤150/>150	83.3/58.1	**0.02**	57.1/50.7	0.63	3.1	1.4-7.6	**0.01**	-	-	-
MUC1, HS median ≤210/>210	44.5/66.8	0.29	44.1/57.3	0.76	-	-	-	-	-	-
MUC1, HS≤150/>150	66.7/55.7	0.85	58.3/48.8	0.83	-	-	-	-	-	-
Mesothelin, HS median ≤150/>150	53.6/62.6	0.39	44.6/58.2	0.36	-	-	-	-	-	-
Mesothelin, HS≤150/>150	53.6/62.6	0.39	44.6/58.2	0.36	-	-	-	-	-	-
MUC4, HS median ≤120/>120	28.6/54.1	0.65	49.8/52.3	0.81	-	-	-	-	-	-
MUC4, HS≤150/>150	53.6/60.3	0.59	49.8/52.3	0.81	-	-	-	-	-	-
Annexin A10, HS median ≤125/>125	49.2/63.1	0.52	46.6/54.9	0.94	-	-	-	-	-	-
Annexin A10, HS≤150/>150	53.2/62.5	0.56	48/55.6	0.89	-	-	-	-	-	-
Glypican-1, HS median ≤50/>50	63.1/52.3	0.35	60.9/44.1	0.22	-	-	-	-	-	-
Glypican-1, HS≤150/>150	58.2/50	0.54	51.3/50	0.93	-	-	-	-	-	-

Supplementary material Figure S1. Kaplan-Meier curve of overall survival (OS) according to CA 19-9 H-score (cut-off 270).

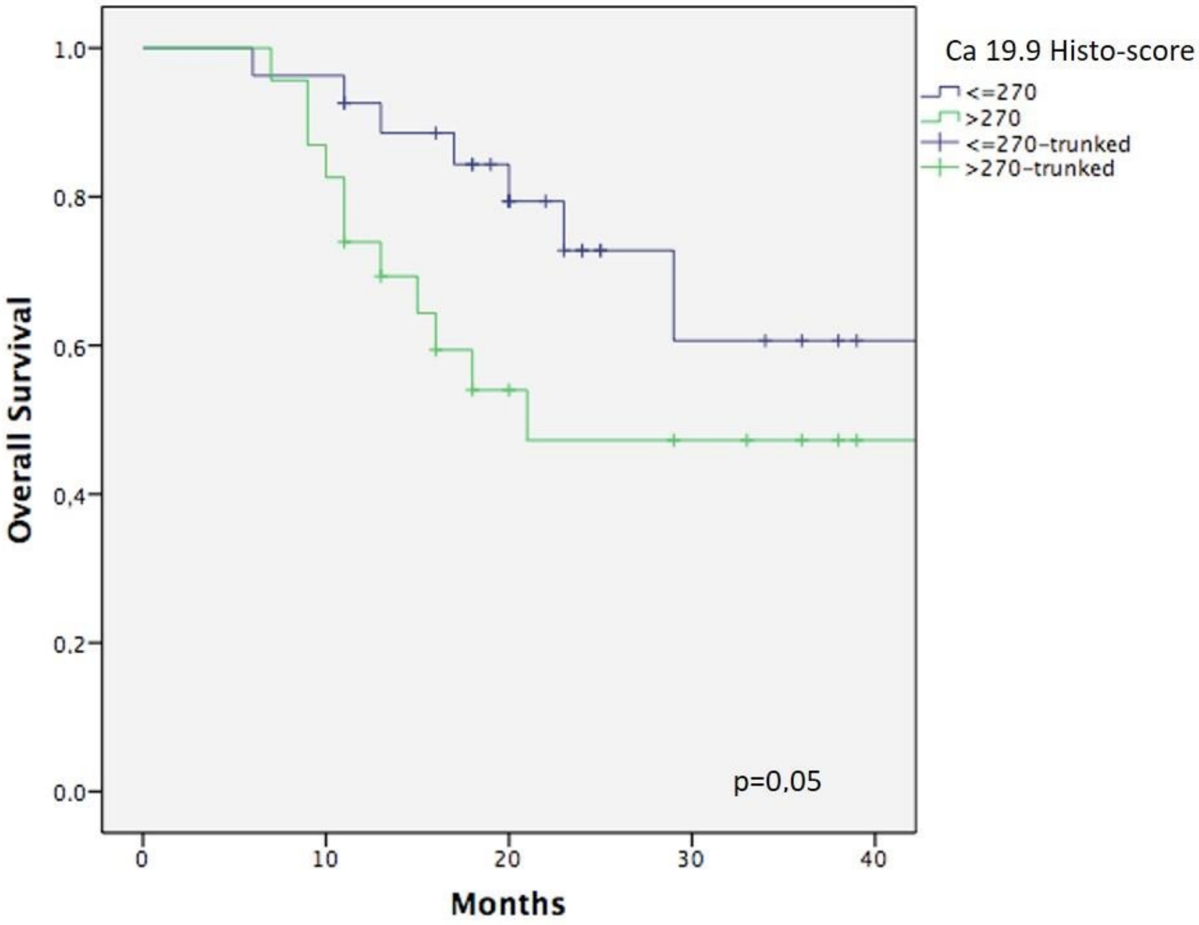

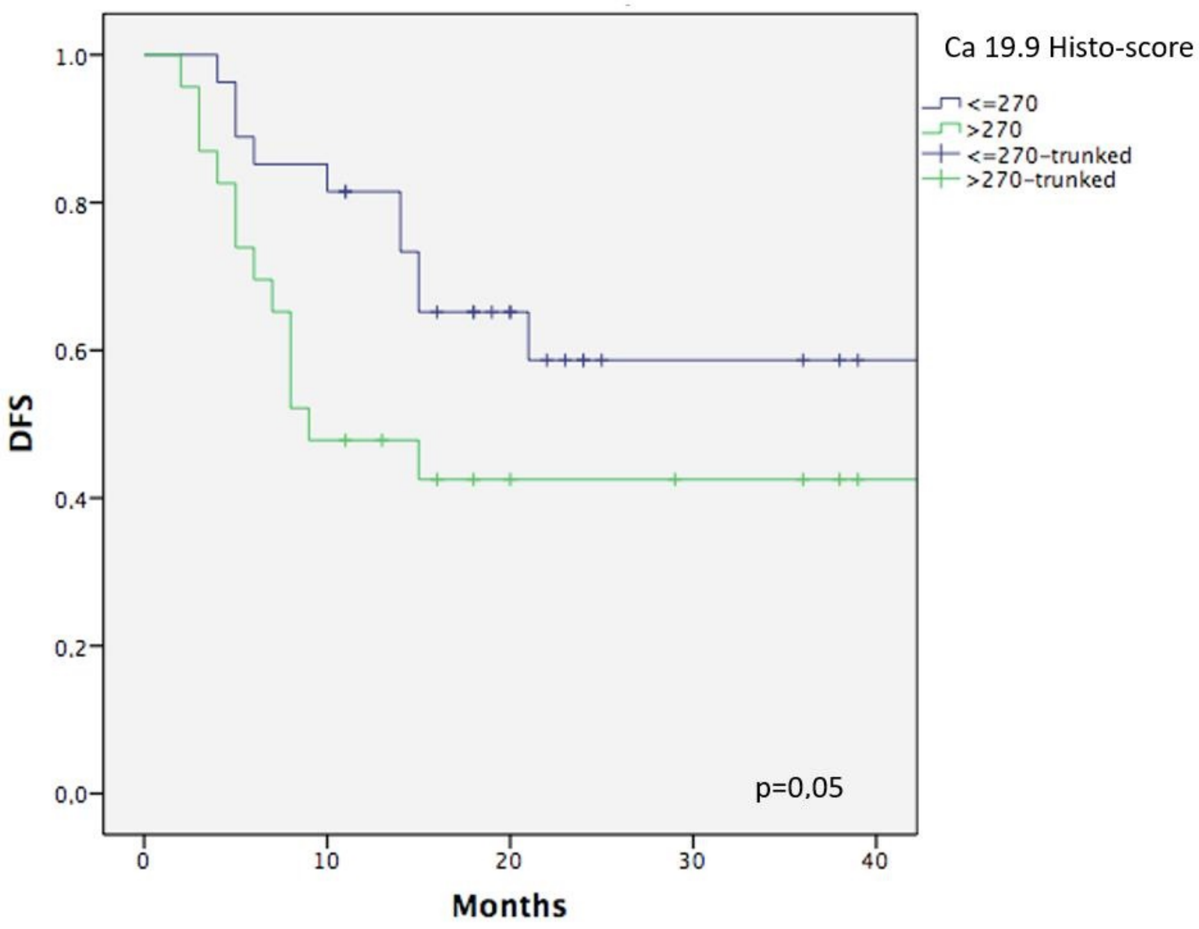

Supplementary material Figure S2. Kaplan-Meier curve of disease-free survival (DFS) according to CA 19-9 H-score (cut-off 270).
